# Supplementary material for: Rapid Generation of Long Noncoding RNA Knockout Mice Using CRISPR/Cas9 Technology
Source: Noncoding RNA. 2019 Jan 23;5(1):12. doi: 10.3390/ncrna5010012 (PMC6468733; doi:10.3390/ncrna5010012)
Supplement: Supplementary file 1 [file ncrna-05-00012-s001.pdf]

Supplementary Figures:

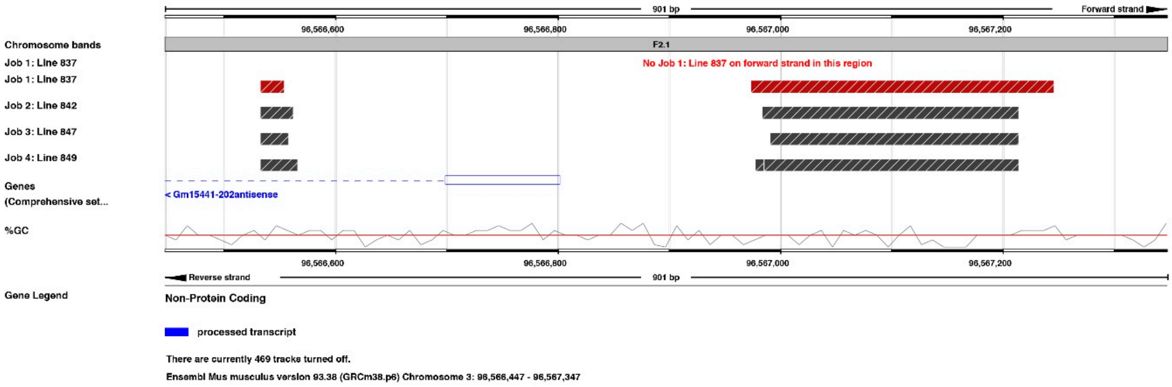

Figure S1: Validated sgRNAs:

Table of sgRNAs that have been evaluated for genome-editing activity, including the crRNA spacer sequence of the respective sgRNA and the outcome of the T7E1 assay. The length of the spacer sequences is indicated in brackets behind the name of the respective sgRNA.

Inheritance of Allele *Gm15541*$^{\Delta 1}$ (Line 837)

Parental Genotypes	Number of Breedings	Total Offspring	Pups per Breeding	Males	Females	Δ/Δ (in %)	wt/Δ (in %)	wt/wt (in %)	x^2-Test (P = 0.05)
F0 X wt/wt	3	29	9.66	13	---	0	23.07	76.93	n.s.
				---	16	0	31.25	68.75	n.s.
wt/Δ X wt/Δ	3	24	8	15	---	20	60	20	n.s.
				---	9	11.11	55.55	33.33	n.s.
wt/Δ X Δ/Δ	1	4	4	0	---	0	0	0	n.s.
				---	4	50	50	0	n.s.

Figure S2: Generated Gm15441-deficient mouse lines:

Overview of Ensemble genome browser tracks depicting *Gm15441*$^{\Delta}$ alleles generated by CRISPR/Cas9-mediated deletion of *Gm15441* exon 1. Error-prone NHEJ repair of Cas9-induced DSBs resulted in minor variations of truncated *Gm15441*$^{\Delta}$ alleles in the four stable *Gm15441*$^{\Delta}$ mouse lines (internal classifications as *Gm15441*$^{\Delta 1}$ [founder #1], *Gm15441*$^{\Delta 6}$ [founder #6], *Gm15441*$^{\Delta 11}$ [founder #11] and *Gm15441*$^{\Delta 13}$ [founder #13]). The deletion size

for the respective mouse lines is as follows: *Gm15441*$^{\Delta1}$, 420 bp; *Gm15441*$^{\Delta6}$, 422 bp; *Gm15441*$^{\Delta11}$, 433 bp; *Gm15441*$^{\Delta13}$, 411 bp. All mice of line *Gm15441*$^{\Delta13}$ harbor an additional T->A base pair substitution at the 3' cutting site (chr3: 96,566,985).

Primer for T7 Endonuclease I Assays	Sequence (5' -> 3')
Gm15441-5'-1 (20) forward	GCTCCTACTCAGACCCTTGTTC
Gm15441-5'-1 (20) reverse	CTCCCTGAGTTGCTTTTGGTC
Gm15441-5'-2 (20) forward	GAAGGGAGATAAAGCGCACG
Gm15441-5'-2 (20) reverse	ATGGGGAGCAAGCCGATAAG
Gm15441-3'-1 (20) forward	GACTAGTCTGATGGAGGCATC
Gm15441-3'-1 (20) reverse	TGTGTGTGTGTGTGAGAGAGAG
Gm15441-3'-2 (20) forward	TCAGCCTGCTTTCTTATATGGC
Gm15441-3'-2 (20) reverse	TGCAAACACAGACATGCACAC
Gm15441-5'-1 (18) forward	GCGCACGTTTAACTGACTCTC
Gm15441-5'-1 (18) reverse	ATAAGCAGCACCCCTCCATG
Gm15441-5'-2 (18) forward	CACAGAAGGGAGATAAAGCGC
Gm15441-5'-2 (18) reverse	TTGCCTTCCCTCACTGATGG
Gm15441-3'-1 (18) forward	ATCAGTGAGGGAAGGCAAGG
Gm15441-3'-1 (18) reverse	AGCAAGCCAGTATCACATGC
Gm15441-3'-2 (18) forward	ATGGAGGGGTGCTGCTTATC
Gm15441-3'-2 (18) reverse	GCAGGAAGGCTAACAGGAGG

Figure S3: Inheritance of Allele Gm15441$^{\Delta}$ *(Line 837):*

Table depicting transgenerational inheritance of the truncated allele *Gm15441*$^{\Delta1}$ in breedings of founder animals (F0) and their progeny. Statistical differences were calculated using chi-squared (x^2)-tests with significance level P=0.05.
